# Multilocus Sequencing of* Corynebacterium pseudotuberculosis* Biotype Ovis Strains

**DOI:** 10.1155/2017/1762162

**Published:** 2017-10-11

**Authors:** Boglárka Sellyei, Krisztián Bányai, Dániel Bartha, István Hajtós, László Fodor, László Makrai

**Affiliations:** ^1^CAR, HAS, Institute for Veterinary Medical Research, P.O. Box 18, Budapest 1581, Hungary; ^2^Government Office for Borsod-Abaúj-Zemplén County, Vologda U. 1, Miskolc 3525, Hungary; ^3^Department of Microbiology and Infectious Diseases, University of Veterinary Medicine, P.O. Box 22, Budapest 1581, Hungary

## Abstract

Thirteen* Corynebacterium pseudotuberculosis* biotype ovis strains isolated from clinical cases of caseous lymphadenitis in Hungary were characterised using multilocus sequencing and their phylogenetic comparison was carried out on the basis of four housekeeping genes (*gro*EL1, *inf*B,* dna*K, and* leu*A). The in silico analysis of the 16 frequently studied housekeeping genes showed that* C. pseudotuberculosis* strains could be readily distinguished from* C. diphtheriae* and* C. ulcerans* strains; however, sequences of the same genes in the two biotypes of the* C. pseudotuberculosis* were highly similar; the heterogeneity values were low. Genes* dna*K, *inf*B,* gro*EL1, and* leu*A showed marked genetic variation within* C. pseudotuberculosis*, and strains of the two biotypes of C*. pseudotuberculosis* could be differentiated. Analysis of the individual genes showed a fairly conservative nature of* C. pseudotuberculosis* biotype ovis strains. The greatest genetic differentiation was seen in the* dnaK* and* infB* genes and concatenations of these two genes were very useful in the genetic separation of the studied strains.

## 1. Introduction

*Corynebacterium pseudotuberculosis *was isolated from a sheep and described by Preisz [1] as a Gram-positive, facultative intracellular pathogen. Two major biotypes can be distinguished based on the ability of reduction of nitrate; the nitrate negative biotype (*C. pseudotuberculosis* biotype ovis) causes caseous lymphadenitis (CLA) in small ruminants [2–4], whereas the nitrate positive biotype (*C. pseudotuberculosis* biotype equi) is the causative agent of ulcerative lymphangitis in horses, cows, camels, buffaloes, and occasionally humans [5, 6]. CLA is a chronic, contagious disease that is characterised by abscess formation in or near major peripheral lymph nodes (external form) or within the internal organs and lymph nodes (internal form). CLA occurs worldwide; however, it is more frequent in the tropical areas and causes important economic losses in ovine and caprine herds by reducing wool, meat, and milk production [4, 7–10].

Classification of* C. pseudotuberculosis* was originally based on cultural, morphological, and biochemical characteristics [11]. In addition to nitrate reduction various molecular methods, restriction fragment length polymorphism (RFLP) of chromosomal DNA, ribotyping, and whole genome sequence analysis were used for the differentiation of* C. pseudotuberculosis* biotypes [12–14].* C. pseudotuberculosis* biotype equi strains showed greater genetic divergence than* C. pseudotuberculosis* biotype ovis strains when examined with pulsed field gel electrophoresis (PFGE), BOX-PCR, random amplified polymorphic DNA (RAPD), and amplification of DNA surrounding rare restriction sites (ADSRRS) [3, 15, 16].

Multilocus sequence typing has become popular in bacterial genotyping technique over the past decade, including genotyping* Corynebacterium* spp.; it allows the identification of potential molecular genetic marker(s) in conservative genes for the differentiation of closely related bacterial strains [17, 18].

The objective of this study was characterisation of Hungarian field strains of* C. pseudotuberculosis* biotype ovis using multilocus sequencing and their phylogenetic comparison on the basis of selected housekeeping genes.

## 2. Materials and Methods

### 2.1. Bacterial Strains and Culture Condition

Thirteen* C. pseudotuberculosis biotype ovis* field strains isolated between 1994 and 2014 were randomly selected from the strain collection of the Department of Microbiology and Infectious Diseases, Faculty of Veterinary Science, Szent István University, Budapest, Hungary. After identification using standard methods [20], they had been stored at −80°C till the examinations. Details about the strains used in this study are described in Table 1.

Reference strains of* C. pseudotuberculosis* biotype ovis (DSM-7180) and* C. pseudotuberculosis* biotype equi (DSM-7177) from the Leibniz Institute, German Collection of Microorganisms, and Cell Cultures (Braunschweig, Germany) were also included in the examinations.

The strains were cultured on Columbia blood agar (LabM, Lancashire, UK) with the addition of 5% (vol/vol) sterile defibrinated sheep blood and incubated at 37°C for 48 h.

### 2.2. In Silico Sequence Analysis and Primer Design

In order to distinguish* C. pseudotuberculosis* biotypes from the closely related species and to find potential markers, the nucleotide sequences of 16 housekeeping genes of 15* C. pseudotuberculosis*, 13* C. diphtheriae* strains, and one* C. ulcerans* strain (Table 2) were compared. The nucleotide sequences of 7 genes including the ATP synthase alpha chain (*atp*A), the DNA polymerase III alpha subunit (*dna*E), the chaperone Hsp70 (*dna*K), the elongation factor G (*fus*A), the 2-isopropylmalate synthase (*leu*A), the 2-oxoglutarate dehydrogenase E1 and E2 components (*odh*I), and the DNA-directed RNA polymerase beta chain (*rpo*B) were taken from [22, 23], while sequences of 9 genes involving ATP synthase beta chain (*atp*D), the heat shock proteins GroEL and GroES (*gro*ES,* gro*EL1, and* gro*EL2), the translation initiation factor IF2 (*inf*B), the DNA recombinational repair system (*rec*A,* rec*N), the alpha subunit of DNA dependent RNA polymerase (*rpo*A), and the manganese-dependent superoxide dismutase (*sod*A) were downloaded from the NCBI database (http://www.ncbi.nlm.nih.gov/genbank/).* In silico* analysis of the selected genes was performed with the ClustalW multiple alignment algorithm [24] in MegAlign software of the Lasergene 7 program suite (DNAStar, Madison, WI, USA) to find potentially useful markers for genetic differentiation and molecular epidemiological investigations.

**Figure 1:**
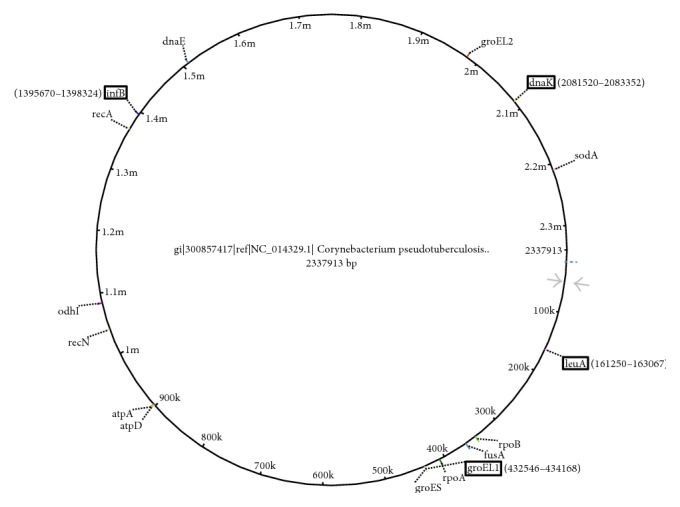
The location of* in silico* studied 16 housekeeping genes at the complete genome map of* C. pseudotuberculosis* FRC41 [19]. The partial regions of rimmed four genes in frames were amplified and sequenced in this study.

Four genes (*gro*EL1, *inf*B,* dna*K, and* leu*A) were selected for primer design; they are located at different genomic regions of bacteria (Figure 1). Primer sets were designed to amplify 580 bp, 654 bp, 641 bp, and 647 bp long fragments from the respective genomic regions; pending on the genes, the number of SNPs varied from 7 to 31 within the amplified fragments (Table 3). PCR primers were designed using Oligo Primer Analysis software 7 [25].

### 2.3. PCR and Sequencing

A loopful of 48 h culture of* C. pseudotuberculosis* was suspended in 6% Chelex-solution (Bio-Rad Laboratories, Hercules, CA, USA) and then heated to 65°C for 30 min and to 100°C for 8 min; afterwards, it was centrifuged. The supernatant was collected in a new tube and stored at −20°C until use. Fragments of the selected housekeeping genes were amplified by PCR in an Applied Biosystems 2720 Thermal Cycler (Thermo Fisher Scientific, Carlsbad, CA, USA). The 25 *μ*l amplification reaction mixture contained 1x Dream Taq Buffer, 200 nM dNTP-mix, 1 *μ*M of each primer, 1 U Dream Taq (Thermo Fisher Scientific, Carlsbad, CA, USA), template DNA, and water. The PCR program included 3 min of initial denaturation at 95°C and then 30 cycles at 95°C for 30 s, at 60°C for 30, and at 72°C for 30 s and 7 min of the final elongation. PCR products were purified for direct sequencing using the Geneaid PCR purification Kit (Geneaid Biotech, New Taipei, Taiwan). Templates were sequenced with the PCR primers using the BigDye Terminator Ready Reaction Mix v3.1. Nucleotide sequences were run on an ABI Prism 3100 Genetic Analyzer (Applied Biosystems, Foster City, CA).

### 2.4. Phylogenetic Analysis

Fragments of four protein-coding highly diverse housekeeping genes (*gro*EL1, *inf*B,* dna*K, and* leu*A) were sequenced for all strains.

Sequences were aligned using the ClustalW algorithm in the MEGA 6 software [26] and trimmed manually at the same position before being used for comparison with sequences of other* C. pseudotuberculosis *strains deposited in the GenBank. The same gene set from* C. diphtheriae* HC02 was used as an outgroup.

After the single gene alignments, the sequences were joined to make a concatemer of loci at head-to-tail in-frame. The phylogenetic trees were constructed by using the neighbour joining (NJ) method. The bootstrap technique [27] was employed to evaluate the reliability of tree topologies by resampling the sequence alignment 1000 times.

### 2.5. Nucleotide Sequence Accession Numbers

The determined partial gene sequences were deposited in GenBank under accession numbers: from MF491615 to MF491629 (*dna*K); from MF464070 to MF464084 (*inf*B); MF476990 and from MF476992 to MF477005 (*gro*EL1); MF446654 and from MF446656 to MF446669 (*leu*A).

## 3. Results

As the use of MLST protocol of* Corynebacterium diphtheriae* (https://pubmlst.org/cdiphtheriae/) proved to be inadequate for analysis of* C. pseudotuberculosis* strains, developing of new scheme has been required.

The* in silico* analysis of the 16 frequently studied housekeeping genes showed that* C. pseudotuberculosis* strains could be readily distinguished by sequence analysis from* C. diphtheriae* and* C. ulcerans* strains; however, sequences of the same genes of the* C. pseudotuberculosis* strains belonging to the two biotypes were highly similar, and the heterogeneity values were low (Table 4). Genes* dna*K, *inf*B,* gro*EL1, and* leu*A showed marked genetic variation within* C. pseudotuberculosis* biotypes and they were selected for further genetic differentiation and molecular epidemiological investigations. The sequence diversity in these regions exceeded 5%.

**Figure 2:**
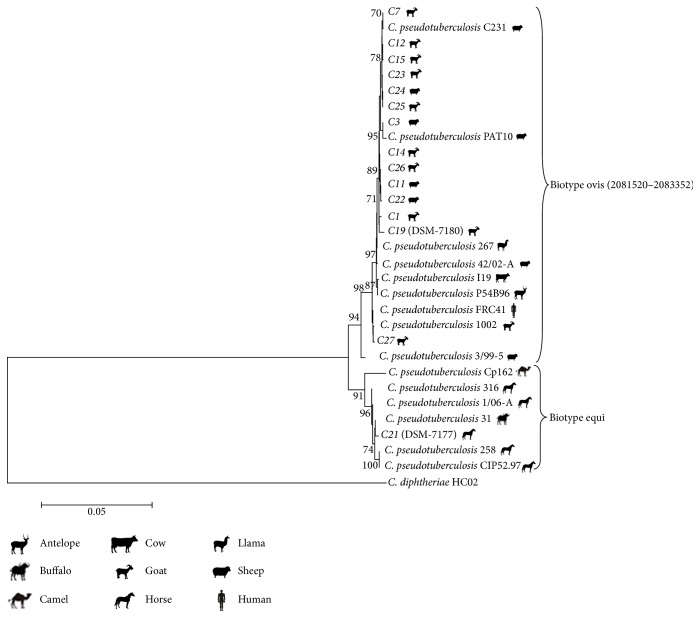
Phylogenetic reconstruction based on the concatenated partial sequence of* dna*K (520 bp), *inf*B (577 bp),* gro*EL1 (588 bp), and* leu*A (597 bp) genes from the Hungarian field isolates and DSM type strains. Analysis was conducted using the neighbour joining (NJ) method. The 1000 bootstrap (BT) values are indicated at branching points. Bar % estimated nucleotide substitutions.

*C. pseudotuberculosis* biotype ovis and* C. pseudotuberculosis* biotype equi field strains were differentiated with sequence analysis of the target gene fragments. Analysis of the individual genes showed a fairly conservative nature of* C. pseudotuberculosis* biotype ovis strains, although segregation of some Hungarian strains (e.g., C1 and C19, C3, C23, and C27) together with strains from Australia, Israel, USA, South Africa, Scotland, France, and Brazil was also evident but they were clearly located in the cluster of* C. pseudotuberculosis* biotype ovis. The greatest genetic differentiation was seen in the* dnaK* and* infB* genes and concatenations of these two genes were very useful in the genetic separation of the studied strains (Figure 2).

## 4. Discussion

*C. pseudotuberculosis* biotype ovis is the causative agent of caseous lymphadenitis, a contagious, chronic disease of sheep and goats [3].

Several previous studies showed high sequential homology of the* C. pseudotuberculosis* biovar ovis strains confirming their clonal origin; however, diversity of the clinical signs in sheep and goats and different efficacy of the vaccines suggest that more than one pathogen clone can exist [14].

The analysis of sequence data of 16 frequently studied housekeeping genes (*atp*A,* dna*E,* dna*K,* fus*A,* leu*A,* odh*I,* rpo*B,* atp*D,* gro*ES,* gro*EL1,* gro*EL2, *inf*B,* rec*A,* rec*N,* rpo*A, and* sod*A) of 15* C. pseudotuberculosis*, 13* C. diphtheriae,* and* C. ulcerans* genomes from the GenBank proved to be related and all studied genes could be used to differentiate* C. diphtheriae, C. ulcerans,* and* C. pseudotuberculosis* strains. All these genes allow the differentiation of biotype ovis and biotype equi strains of* C. pseudotuberculosis* but for detailed examination of* C. pseudotuberculosis* biovar ovis field isolates four genes (*dna*K,* gro*EL1, *inf*B, and* leu*A) seemed to have higher differentiation power. The partial sequence analysis of adequate genes delineated phylogenetic trees with various topology and depth of branches. The highest resolution and topology of the tree were obtained with the* dna*K sequences.

The clusters could be explained by movement of breeding animals, but no epidemiological connection was found between the examined* C. pseudotuberculosis* strains. The distance between the geographical place of isolation and the year of the isolation do not support any epidemiological connection between the strains, so circulation of several clones of* C. pseudotuberculosis* biotype ovis was verified.

According to our data, multilocus sequence typing can differentiate* C. pseudotuberculosis* and the most important* Corynebacterium* species, and examination of selected genes helps to differentiate the two biotypes of* C. pseudotuberculosis*, and it can be used for epidemiological follow-up of these strains.

## Figures and Tables

**Table 1:** The list and registered data of *C. pseudotuberculosis* strains included in the examinations.

ID	Host	Place of isolation	Strains	Year
C1	Goat	Szentistván	*C. pseudotuberculosis* biotype ovis	1994
C3	Sheep	Vizsoly	*C. pseudotuberculosis* biotype ovis	2003
C7	Goat	Vizsoly	*C. pseudotuberculosis* biotype ovis	2003
C11	Sheep	Boldogkőváralja	*C. pseudotuberculosis* biotype ovis	2005
C12	Goat	Érd	*C. pseudotuberculosis* biotype ovis	2005
C14	Goat	Alsózsolca	*C. pseudotuberculosis* biotype ovis	2006
C15	Goat	Rudabánya	*C. pseudotuberculosis* biotype ovis	2006
C19	Goat	(DSM-7180)	*C. pseudotuberculosis* biotype ovis	1988
C21	Horse	(DSM-7177)	*C. pseudotuberculosis* biotype equi	1988
C22	Sheep	Mezőcsát	*C. pseudotuberculosis* biotype ovis	2013
C23	Goat	Csetény	*C. pseudotuberculosis* biotype ovis	2013
C24	Sheep	Pétervására	*C. pseudotuberculosis* biotype ovis	2009
C25	Goat	Szendrő	*C. pseudotuberculosis* biotype ovis	2011
C26	Goat	Csongrád	*C. pseudotuberculosis* biotype ovis	2014
C27	Goat	Kisvásárhely	*C. pseudotuberculosis* biotype *ovis*	2014

**Table 2:** The source of *Corynebacterium* sp. examined in the in silico sequence analysis.

*C. pseudotuberculosis* ^&^	Biotype	Host	Origin	*C. diphtheriae* ^§^	Host	Origin
FRC41	Ovis	Human	France	NCTC 13129	Human	UK
3/99-5	Ovis	Sheep	Scotland	BH8	Human	Rio de Janeiro
P54B96	Ovis	Antelope	South Africa	C7	Human	Atlanta, GA, USA
1002	Ovis	Goat	Brazil	241	Human	Rio de Janeiro
C231	Ovis	Sheep	Australia	HC01	Human	Rio de Janeiro
I19	Ovis	Cow	Israel	VA01	Human	Rio de Janeiro
PAT10	Ovis	Sheep	Argentina	CDCE 8392	Human	Bethesda, MD, USA
42/02-A	Ovis	Sheep	Australia	31A	Human	Rio de Janeiro
267	Ovis	Llama	USA	HC03	Human	Rio de Janeiro
316	Equi	Horse	USA	HC02	Human	Rio de Janeiro
CIP 52.97	Equi	Horse	Kenya	INCA 402	Human	Rio de Janeiro
1/06-A	Equi	Horse	USA	PW8	Human	New York
31	Equi	Buffalo	Egypt	HC04	Human	Rio de Janeiro
258	Equi	Horse	Belgium	*C. ulcerans* ^#^		
Cp162	Equi	Camel	UK	BR-AD22	Dog	Rio de Janeiro

^&^[14]; ^§^[19]; ^#^[21].

**Table 3:** Primer sequences used for amplification and sequence analysis.

Locus	Putative gene product	Primer name	Sequence	Product size (bp)	Position on NC_014329.1 (FRC41 strain)
*dna*K	Chaperone Hsp70	Cps dnaK F	5′-TCCTTACCAGTGCCCTTATCC-3′	580	2081944–2081964
		Cps dnaK R	5′-GAGTTCCAGCGCATCACC-3′		2082507–2082524
*gro*EL1	Heat shock protein	Cps groEL1 F	5′-ACCTTCACCGGATCATTG-3′	654	1978987–1979004
		Cps groEL1 R	5′-TTGGTGATCGTCGTAAAGC-3′		1979623–1979641
*inf*B	Translation initiation factor IF2	Cps infB F	5′-ATTGCGGGACTTGGACG-3′	641	1397768–1397784
		Cps infB R	5′-GCATTATGCTGCACAAGACG-3′		1398390–1398409
*leu*A	2-isopropylmalate synthase	Cps leuA F	5′-AGCTCAGTGCGCGGTTGACC-3′	647	161252–161271
		Cps leuA R	5′-ATGGCGTCGCGGGTTCG-3′		161915–161899

**Table 4:** Nucleic acid average sequence divergence of housekeeping genes in *Corynebacterium* species in the *in silico* study.

Gene	Full length (bp)	Average sequence divergence		
		*C. diphtheriae* versus *C. pseudotuberculosis*	*C. ulcerans* versus *C. pseudotuberculosis*	*C. pseudotuberculosis* biotype ovis versus equi
*gro*EL1	1641	62.9	27.9	1.1
*inf*B	2886	13.6	6.0	0.8
*dna*K	1836	9.1	5.0	0.6
*leu*A	1818	14.0	8.1	0.5

*odh*I	3687	12.5	49.7	0.4
*atp*D	1446	6.2	3.2	0.4
*rec*N	1740	27.0	10.6	0.4
*dna*E	3561	58.2	7.1	0.3
*gro*EL2	1641	59.5	27.6	0.3
*rec*A	1110	14.1	7.3	0.3
*rpo*A	1017	7.7	3.5	0.3
*fus*A	2127	3.9	2.6	0.2
*gro*ES	297	6.5	3.5	0.2
*rpo*B	2974	8.0	3.3	0.2
*sod*A	600	10.0	4.3	0.2
*atp*A	1630	8.4	2.1	0.0
